# Extramedullary Plasmacytoma Presenting as a Solitary Mass in the Intracranial Posterior Fossa

**DOI:** 10.5812/iranjradiol.8759

**Published:** 2012-11-20

**Authors:** Mohammad Hossein Daghighi, Masoud Poureisa, Mohammad Shimia, Ramin Mazaheri-Khamene, Shadi Daghighi

**Affiliations:** 1 Department of Radiology, Radiotherapy and Nuclear Medicine, Tabriz University of Medical Sciences, Tabriz, Iran; 2 Neurosciences Research Center (NSRC), Tabriz University of Medical Sciences, Tabriz, Iran; 3 Department of Neurosurgery, Shohada Hospital, Tabriz University of Medical Sciences, Tabriz, Iran; 4 Department of Clinical Science, Veterinary Faculty, Urmia University of Medical Sciences, Urmia, Iran; 5 Tabriz University of Medical Sciences, Tabriz, Iran

**Keywords:** Plasmacytoma, Magnetic Resonance Imaging, Posterior Fossa

## Abstract

A patient with a 3-month history of headache refractory to pain medication was admitted. The CT scan and MRI showed evidence of a posterior fossa mass. This was pathologically confirmed as an extra medullary plasmacytoma (EMP). He had a pathologic fracture of the left humerus 7 years ago while the radiologist was unaware at the time of diagnosis. A solitary bone plasmacytoma (SBP) was the cause of the pathologic fracture. This report includes the first description of MRI findings in a patient with a rare-incidence intracranial solitary extra medullary plasmacytoma (SEP) in Iran. There is a striking similarity between the features of intracranial SEP and meningiomas. Intracranial SEP, although rare, should be included in the differential diagnosis of brain tumors in areas where meningiomas commonly arise. The MRI findings and differential diagnosis of plasmacytoma are reviewed. Before this case report, only few cases have been reported in the literature. Nonetheless, this is the first report of posterior fossa EMP from Iran.

## 1. Introduction

Plasmacytoma is a discrete, solitary mass of neoplastic monoclonal plasma cells in either the bone or soft tissue (extramedullary). The skull and central nervous system are rarely involved by plasma cell tumors without evidence of plasma cell dyscrasia at another site (1). The EMP represents approximately 3% of all plasma cell neoplasms. The median age of presentation of EMP is 55 years and 75% of the patients are men; 80% is localized in the head and neck region (nose, sinuses, and the nasopharynx). Other locations are the gastro-intestinal tract, lungs, breasts, testes and the skin. This tumor presents as a mass growing in the aerodigestive tract in 80-90% of the patients, often spreading to the lymph nodes, although other sites may be affected as well. Solitary extramedullary plasmacytoma (SEP) accounts for less than 2% of all neoplastic plasma cell dyscrasias and it may occur in any part of the body (2). Nevertheless, this rare tumor represents only less than 1% of all malignancies in the head and neck region (3, 4). Symptoms of EMP in other tissues are associated with the site of the tumor, tumor size and compression and/or involvement of the surrounding structures (5). The imaging appearance of solitary plasmacytoma can simulate more common neoplasms such as meningiomas. The treatment of EMP consists of radiotherapy and/or surgery. The prognosis after radical surgical resection is similar to radiotherapy. Many consider the combination of surgery and radiotherapy as the treatment of choice (3). This case is the first report of intracranial posterior fossa EMP in the Iranian population.

## 2. Case Presentation

**Figure 1:**
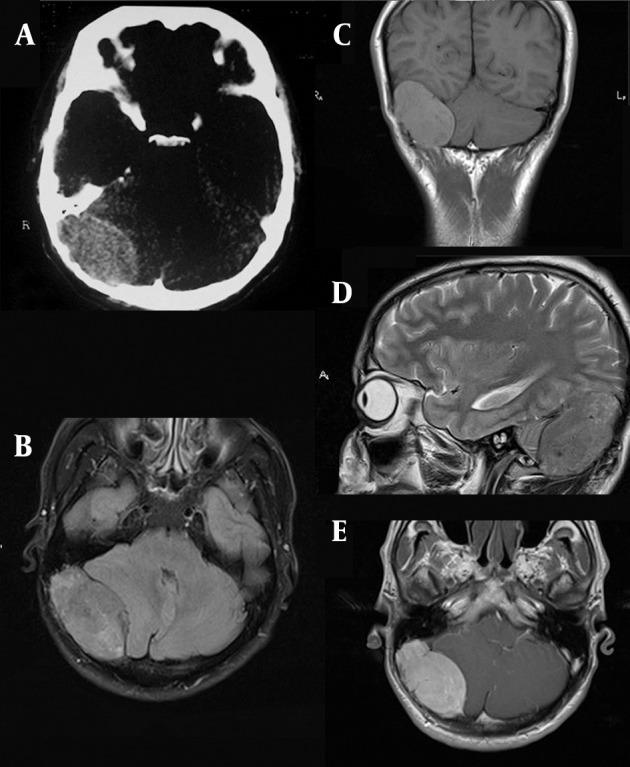
A 37-year-old man with headache, large extraaxial iso-signal space occupying lesion on the right side of the posterior fossa with erosion of the internal table and compressive effect to the right cerebellar hemisphere and marked enhancement. A, CT image with erosion of the internal table; B, Axial FLAIR MRI with isosignal extra axial SOL at the right side of the posterior fossa; C, Coronal T1 WI without contrast; D, Sagittal T2 WI; E. Axial post-contrast T1 WI.

A 37-year-old man was admitted in May 2011 with a 3-month history of headache refractory to pain medication. He had periodic medical examinations and displayed no abnormalities until the occipital headache began 3 months prior to admission. He denied a history of fatigue. Neurological examination was normal. CT revealed an extra axial mass on the right side of the posterior fossa. There was erosion of the inner table of the skull in CT scan images (Figure 1A). In the magnetic resonance imaging (MRI) (1.5 T) an isointense to gray matter mass, 55 × 35 × 31 mm in size was detected on FLAIR, T1 and T2 weighted images (WI) (Figure 1B-D). The vermis and the fourth ventricle were displaced to the left side and minimal hydrocephalus was also present. The mass enhanced severely after the injection of contrast media (Figure 1E). It had a similar appearance to meningioma. In addition, the site of the incidence was the location where meningioma commonly arises. Meningioma (the most probable), intracranial multiple myeloma (MM) and lymphoma were considered in the initial differential diagnosis list. Hospital admission laboratory workup revealed no hematological and biochemical abnormalities. Liver and kidney function tests were also unremarkable. He underwent surgery for the posterior fossa lesion. Suboccipital approach craniotomy was performed.

**Figure 2:**
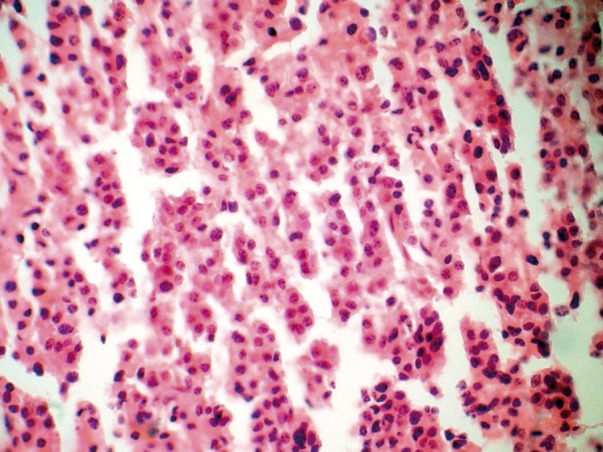
Diffuse infiltration of medium-sized plasma cells with the characteristic of mild pleomorphic round oval cells, eccentric nuclei and moderate cytoplasm on H&E staining indicating a plasmacytoma.

Postoperatively, the patient was alert and showed no additional neurological deficit. Histopathological study with H&E staining demonstrated diffuse infiltration of medium-sized plasma cells with the characteristic of mild pleomorphic round oval cells, eccentric nuclei and moderate cytoplasm (Figure 2). Areas of necrosis were also present in serial sections. There was also an increased nuclear to cytoplasmic ratio and some cells had prominent nucleoli. For definite diagnosis and rule out of lymphoma, immune histochemical (IHC) staining with CD45 was considered. The infiltrating cells showed negative staining with CD45. It confirmed a diagnosis of plasma cell tumor. Bone marrow (BM) aspiration at the time of diagnosis showed no evidence of plasmacytosis. Urine studies for Bence Jones proteins, serum protein electrophoresis and urine protein electrophoresis were negative. He was discharged on a steroid regimen with a plan for radiation therapy. He had a pathological fracture of the left humerus 7 years ago. The definitive pathology report of the humeral lesion confirmed the diagnosis of solitary bone plasmacytoma (SBP), but the radiologist was unaware about this occurrence till the pathology report confirmed the nature of the mass as an SEP.

## 3. Discussion

Plasmacytoma is referred to a single lesion without any evidence of MM in any other part of the body (6, 7). Intracranial plasma cell tumors are rare, with only isolated case reports and small series in the literature. There are several reports of plasma cell tumors involving the cranium, while we found only four other reports of plasma cell dyscrasia manifesting as a mass in the posterior fossa(8, 9).

Solitary craniocerebral plasmacytoma is a separate entity from the far more common plasma cell tumor that arises within the cranium as a result of disseminated MM. As mentioned above, previous BM aspiration repudiated the diagnosis of MM, as well as he had none of the classic symptoms of systemic disease. In solitary plasmacytoma, plasma cell monoclonal proliferation is localized in the bone marrow; therefore, bone pain, bone destruction and pathological fractures represent the most common clinical sign of the disease (10). Moreover, bone damage may also be responsible for alteration of blood calcium levels, but this alteration is more frequent in multiple myeloma than in solitary plasmacytoma (10). As seen in our case, there was no frank alteration of blood calcium levels. Some typical findings of a dural and/or osseous plasmacytoma include isodensity to hyperdensity on CT scan, T1 equal to high signal intensity and T2 markedly hypointense signal on MRI and high vascularity possibly documented on digital subtraction angiography (11). In this case, the mass was isointense to gray matter both on T1 and T2 WI. Previous reports of the MR imaging appearance of solitary plasmacytoma of the skull have described a slightly inhomogeneous, expansile mass eroding the bone that is isointense to the brain on noncontrast T1 WI and isointense or slightly hyperintense on T2 WI, (12, 13) which were similar to the findings in our patient. The extraaxial location, sharp margins between the tumor and the brain, signal characteristics and the enhancement pattern of the lesion in our case bore some similarities to meningioma. Several studies and case reports have established the imaging similarities between plasmacytoma and meningiomas (12, 14). However, the neuroradiological findings generally lack specificity, since they are generally no different from those of meningioma, metastasis, lymphoma, dural sarcoma, plasma cell granuloma, infectious meningitis and leptomeningeal carcinomatosis (11).

According to the most recent World Health Organization classification, plasma cell neoplasms can be divided into extramedullary plasmacytoma, which include malignant plasma cell tumor and plasmacytoma, and multiple myeloma, also known as plasma cell myeloma. These represent a spectrum of the disease, where plasmacytoma refers to the localized disease and multiple myeloma implies systemic dissemination. Plasmacytoma, however, can progress to MM (15, 16). In our patient, as mentioned above, neither the results of bone marrow biopsy nor IHC staining with CD45 or classic symptom of systemic disease confirmed the diagnosis of MM or lymphoma.

Plasmacytoma is a highly radiosensitive tumor. All cases of solitary plasmacytomas of the calvarium reported in the literature have been treated by surgery and radiotherapy. Several authors stress that its sufficient diagnosis and treatment with conventional external radiotherapy may be satisfactory (1, 3, 10, 16, 17). Periodic evaluation for progression and development of MM, SBP and EMP is recommended, with planning of clinical appointments thereafter. Besides, a complete history and physical examination, complete blood cell (CBC) count, complete metabolic panel with lactic dehydrogenase (LDH), calcium, phosphorus, C-reactive protein (CRP), and beta2 microglobulin, erythrocyte sedimentation rate (ESR), serum protein electrophoresis with immunofixation, serum immunoglobulin quantification, urinary protein electrophoresis with immunofixation (24-h urine sample) and skeletal bone survey are recommended (16).

We present a patient with rare SEP as a large mass in the posterior fossa that highlights the importance of plasma cell tumors in the differential diagnosis of intracranial masses such as meningioma. These lesions share the same imaging findings on both CT scan and MRI. Epidemiological factors and the location of the lesion can help, but the final diagnosis is only confirmed by histological examination. Additionally, it is critical for the patient to receive a full systemic work-up to evaluate if the patient has MM. Despite this, an accurate clinical examination and perfect history taking by the radiologist must be considered as a rule.
